# The evolution and significance of embolization efficiency index (EEI) during transcatheter hepatic arterial embolization procedure: a pilot study based on computational fluid dynamics

**DOI:** 10.3389/fbioe.2025.1638266

**Published:** 2025-11-25

**Authors:** Zi-Xuan Wang, Yi-Fan Zhao, Quan Qi

**Affiliations:** 1 Department of Interventional Radiology, Qingdao Hospital, University of Health and Rehabilitation Sciences (Qingdao Municipal Hospital), Qingdao, Shandong, China; 2 Joint Innovation Laboratory for Intelligent Interventional Procedures, Qingdao Hospital, University of Health and Rehabilitation Sciences (Qingdao Municipal Hospital), Qingdao, Shandong, China; 3 School of Information Science and Technology, Shihezi University, Shihezi, Xinjiang, China

**Keywords:** embolization efficiency index, transcatheter arterial embolization, interventional radiology, hepatic artery, computational fluid dynamics

## Abstract

**Purpose:**

To investigate the hemodynamic determinants of the embolization efficiency index (EEI) during transcatheter hepatic arterial embolization (TAE), with the goal of improving embolization protocols, optimizing therapeutic precision, and mitigating the risks of non-target embolization.

**Methods:**

Using computational fluid dynamics and patient-specific right hepatic artery geometry from cone-beam CT angiography, we evaluated the impact of inlet flow rate, target outlet pressure, and vascular hierarchy on EEI. Simulations in OpenFOAM solved Navier-Stokes equations under steady and pulsatile flow.

**Results:**

There was no correlation between inlet flow rate and EEI. Instead, EEI exhibited an inverse linear relationship with target pressure, declining until flow cessation at a certain pressure greater than 5000Pa. Proximity to the target outlet significantly enhanced EEI. Backflow phenomenon indicated inherent non-target embolization risks under pulsatile conditions.

**Conclusion:**

This proof-of-concept study suggests that, in this model, EEI is primarily influenced by outlet pressure and vascular anatomy, rather than inflow dynamics. These findings call into question the conventional emphasis on flow modulation, pointing to the potential value of pressure-aware strategies and superselective catheter placement near targets. Based on the analyzed case, the study offers quantitative thresholds and spatial EEI gradients that could help refine TAE precision and potentially reduce complications. With further validation, integrating such CFD-based EEI metrics into procedural planning may contribute to standardizing embolization protocols.

## 1 Introduction

Transcatheter arterial embolization (TAE) is a cornerstone in the management of hepatocellular carcinoma (HCC) and metastatic liver tumors, leveraging targeted occlusion of tumor-feeding arteries to induce ischemic necrosis (Castiglione et al., 2025; Lanza et al., 2025). Despite its clinical efficacy, suboptimal embolic distribution influenced by hemodynamic complexity remains a challenge, potentially leading to incomplete tumor response or off-target complications (Moon et al., 2023; Gowda et al., 2021; van Roekel et al., 2021). Embolization efficiency, defined as the proportion of embolic agents reaching the target vasculature, is critical for therapeutic success but remains poorly quantified in dynamic vascular environments.

Recent advances in computational fluid dynamics (CFD) have enabled detailed simulations of arterial hemodynamics, offering insights into flow patterns that influence embolic trajectories (Corti et al., 2021). Previous studies (Taebi et al., 2020a; Aramburu et al., 2022; Aramburu et al., 2016) demonstrated the utility of CFD in modeling hepatic arterial flow, emphasizing the role of vascular geometry and boundary conditions. However, the interplay between hemodynamic parameters and embolization efficiency remains underexplored. Traditional metrics like vascular embolization endpoints (e.g., contrast agent stasis) lack quantitative rigor (Periyasamy et al., 2021), underscoring the need for a standardized indicator, such as the embolization efficiency index (EEI).

Prior research highlights the impact of outlet pressure on flow redistribution post-embolization. For instance, Vikström et al. (2024) identified resistance boundary conditions as critical determinants of flow partitioning in arterial networks, while Roncali et al. (2020) correlated CFD-predicted flow patterns with clinical outcomes in radioembolization. Despite these strides, gaps persist in understanding how dynamic flow variations and anatomical hierarchies modulate EEI.

This study introduces a novel CFD-based framework to evaluate EEI during TAE, addressing two key questions: (1) How do inlet flow rate and target outlet pressure influence EEI? (2) How does vascular anatomical hierarchy affect embolic distribution? By integrating patient-specific cone beam computed tomography (CBCT) angiography and OpenFOAM-based simulations, we analyze hemodynamic responses under varying pressure and pulsatile flow conditions. Our findings aim to refine embolization protocols, optimizing therapeutic precision while mitigating risks of non-target embolization.

## 2 Materials and methods

### 2.1 Subjects

The subject is a 62-year-old male patient from the interventional radiology department. His right hepatic artery has been identified as healthy and included in the study. The protocol has been approved by the clinical trial ethics committee/institutional review board (IRB) (2024-KY-033), and informed written consent was obtained from the patient.

### 2.2 CBCT angiograph

**FIGURE 1:**
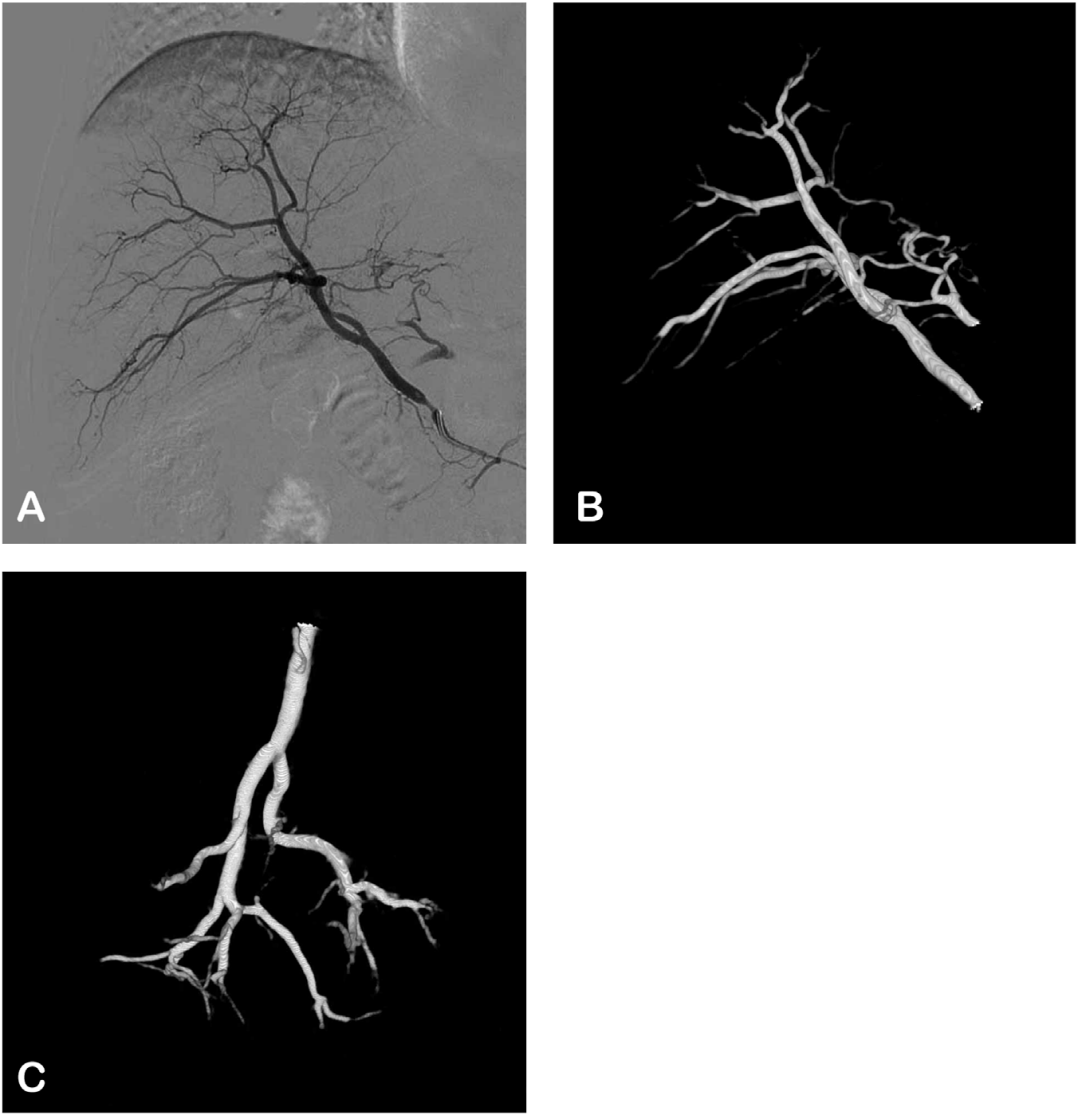
The angiographic images of the right hepatic artery in a 62-years-old male. **(A)** Digital subtraction angiography (DSA): postero-anterior view. **(B,C)** 3D views based on CBCT angiography.

The procedure was conducted with a CBCT-capable angiography system (UNIQ FD 20, Philips Healthcare, Best, Netherlands). Hepatic artery angiography via the femoral artery approach was performed using a 5 French RH angiographic catheter (Cordis Corporation, Miami lakes, FL, United States) (Figure 1A). The Xper CT abdomen HQ protocol involved acquiring 620 projections by rotating the C-arm 240° around the patient for 5.2 s. The imaging parameters were set at 120 kV and 265 mA, with a field of view (FOV) matrix size of 512 × 512. The scan under breath-hold began 3 s after injecting 21 mL of contrast medium (320 mg Iodixanol, Jiangsu Hengrui Pharmaceuticals Co., Ltd., Lianyungang, China) through a RH catheter in the right hepatic artery at a rate of 4 mL/sec. The injector used was the Mark V ProVis Injector (MedradInc., Indianola, PA, United States) (Figures 1B,C).

### 2.3 Segmentation and meshing

**FIGURE 2:**
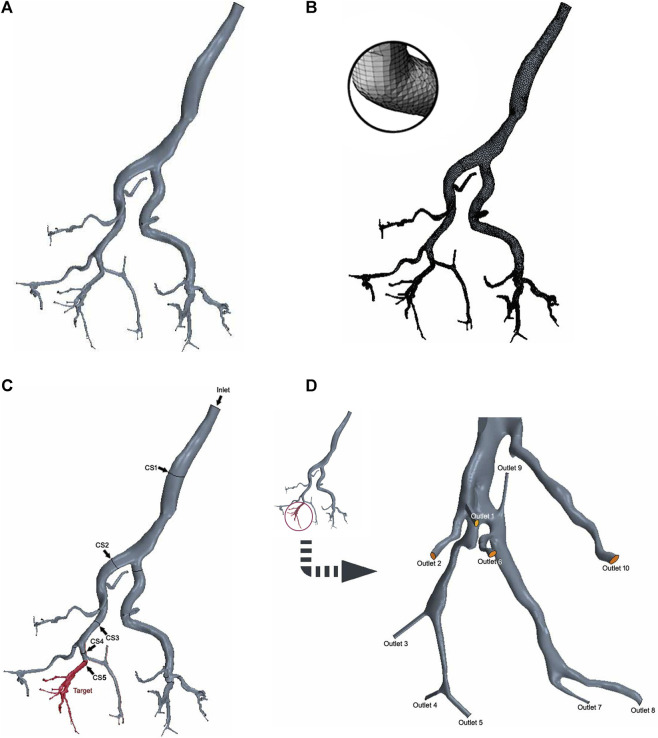
3D digital right hepatic arterial tree in CFD simulations. **(A)** 3D simulation geometries. **(B)** Mesh generation. **(C)** Cross-sections 1–4 (arrow) and the target branch (red). **(D)** Target outlets 1-10.

The DICOM files from CBCT angiography were imported into the 3D Slicer software (Harvard, Boston, United States, https://www.slicer.org/) to generate the geometric 3D model in. stl format. Subsequently, the model was imported into the snappyHexMesh tool integrated within the OpenFOAM platform (OpenFOAM Foundation Ltd., United Kingdom) for segmentation and meshing of the hepatic arterial tree. The 3D reconstructions of the arterial trees underwent additional processing, including smoothing and truncating the outlets, resulting in the 3D simulation geometries displayed in Figure 2A.

The mesh size was determined based on previous CFD studies’ meshing expertise and the results of a preliminary grid independence study, leading to the generation of high-quality surface meshes (Taebi et al., 2020a). The final linear tetrahedral mesh comprised approximately 400,000 elements, with increased mesh densities strategically placed at bifurcations and boundaries to effectively capture more intricate flow patterns (Figure 2B).

### 2.4 Hemodynamic governing equations and parameters

According to previous literature reports, the governing hemodynamic equations used to solve the laminar incompressible blood flow in the hepatic artery include the three-dimensional Navier–Stokes conservation of mass and momentum equations (Taebi et al., 2020b) as follows (Equations 1, 2).

$$\nabla .\left(u\right)=0.$$ (1)

$$\rho \left(\frac{\partial u}{\partial t}+u.\nabla u\right)=-\nabla p+\mu \nabla^{2}u+\frac{1}{3}\mu \nabla \left(\nabla .u\right).$$ (2)

In the aforementioned equations, u represents the flow velocity, p denotes flow pressure, *ρ* indicates blood density, and μ signifies dynamic viscosity. For the purposes of this study on the hepatic artery, the blood flow behavior is analyzed using a Newtonian model. Therefore, it is assumed that the blood is both incompressible and Newtonian, with a density and dynamic viscosity of 1,060 kg/m^3^ and 0.0035 Pa⋅s, respectively. Subsequent calculations reveal that the maximum Reynolds number (Re = 417) in this study is below 2,300, thus indicating a laminar flow state. All of the hemodynamic equations for blood flow in the hepatic artery were numerically solved using the finite volume scheme in the OpenFOAM platform.

### 2.5 Boundary conditions

Since the left lobe has been surgically removed, it was not included in the computational domain. Therefore, the computational model only consisted of the hepatic arterial tree in the right lobe with an inlet at the right hepatic artery (Figure 2C).

The hepatic arterial tree analyzed in this study had one inlet and 43 outlets. All branches are at a certain depth level (n = 1, … , N). The depth level of an artery is determined by the number of branches the blood has passed through after passing through that artery, starting from the inlet branch of the domain where the depth level is one. In this arterial tree, one branch (n = 5) was designated as the target branch for embolization (Figure 2C). The target branch further divides into 10 target outlets (Figure 2D, outlets 1-10). Along the path from the inlet to the target branch, a cross-section is established at the midpoint of each branches (Figure 2C, CS 1-5).

**FIGURE 3:**
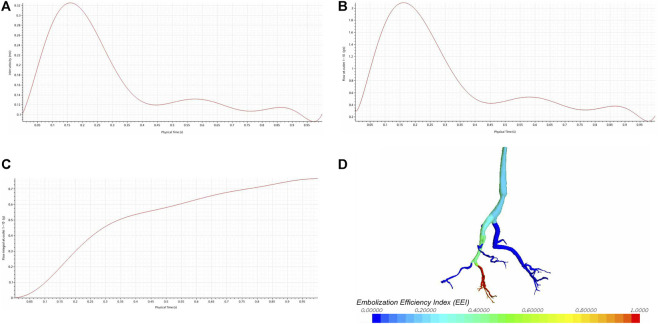
The impact of different inlet flow rates on the embolization efficiency index (EEI). **(A)** The inlet velocity curve. **(B)** The outlet flow rate curve. **(C)** The integral results of the outlet flow rate. **(D)** The EEI cloud map.

All parameters in this study were obtained from previous research, human physiological data, or pre-experiments. As the study examined the impact of different inlet flow rates on the embolization efficiency index, a representative pulsatile waveform (Figure 3A) with a parabolic velocity profile was approximated and adjusted based on published data (Aramburu et al., 2022). The outlet pressure is set to 300 Pa. When studying the stable flow conditions, the flow rate at the inlet was 5 cm^3^/s. To investigate the impact of outlet pressure on hepatic artery hemodynamics and flow distribution, simulations were conducted by varying the total pressure of the target branch from 0 Pa to 5,000 Pa (Six conditions, including 0 Pa, 1,000 Pa, 2000 Pa, 3,000 Pa, 4,000 Pa, and 5,000 Pa, were included.). The pressure variation range in the target branch falls within the normal physiological parameter thresholds, and its rationality has been demonstrated by the analysis results. Specifically, when the pressure is 0 Pa, the outlet flow rate reaches its maximum. As the pressure increases, the outlet flow rate gradually decreases. When the pressure reaches 5,000 Pa, the outlet flow rate approaches 0 cm^3^/s, which resembles the hemodynamic condition during vascular or hepatic sinusoid occlusion. Higher pressures would result in reverse flow, which is inconsistent with the anatomical and physiological reality of the hepatic artery. This total pressure was then distributed to each outlet (outlets 1-10) considering a direct proportional relationship with the outlet cross-sectional areas. All simulation scenarios assumed a rigid wall with no-penetration and no-slip conditions.

### 2.6 Study outcomes

The Embolization Efficiency Index (EEI) is defined as the proportion of the quantity flowing into the target branch when an embolic agent is injected at a specific location in the arterial tree relative to the total volume. This is approximately equal to the ratio of the flow into the target branch passing through the embolization operation site to the total flow at the embolization operation site (Equation 3).

$$\text{EEI}=\frac{\sum Q_{target}}{Q_{total}}\times 100\%$$ (3)

Where Q_target_ is the target outlet flow rate and Q_total_ is the total flow rate. Other study outcomes include inlet flow rate, cross-sectional flow rate, outlet flow rate, and outlet pressure.

## 3 Results

Figure 3B shows the flow rate curve at the CS 5, which represents the summed flow rates from outlets 1-10. The shape of the outlet flow rate curve mirrors that of the inlet flow rate curve (Figure 3B), indicating a positive correlation. The integral results of the outlet flow rate are displayed in Figure 3C. Despite variations in the inlet flow rate, there is no corresponding change in the EEI cloud map (Figure 3D), suggesting no correlation between the inlet flow rate and EEI.

**FIGURE 4:**
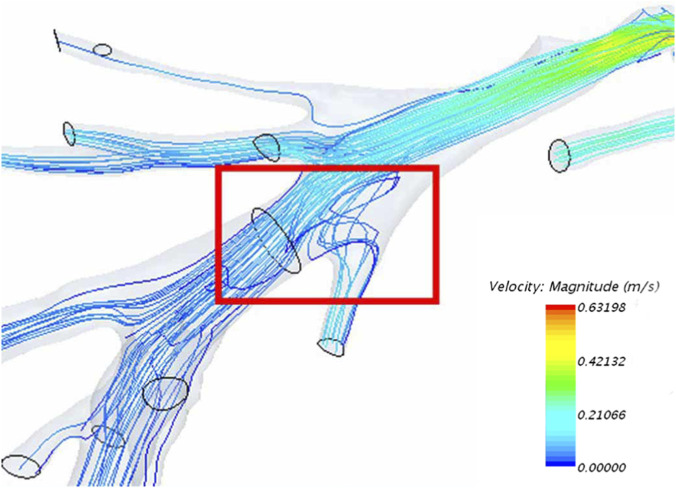
The backflow phenomenon in the flow field (red square).

As a result of factors such as vascular anatomical structure, backflow is an inevitable phenomenon experienced by blood flow within the vessels (Figure 4).

**FIGURE 5:**
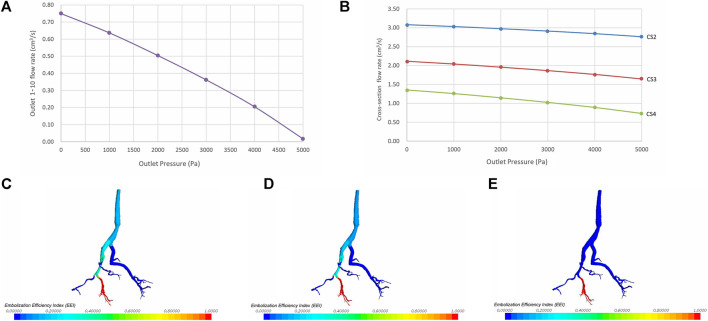
The impact of varying target outlet pressures on the embolization efficiency index (EEI). **(A)** Correlation between outlet pressure and flow rate. **(B)** Correlation between outlet pressure and flow rates at cross-section 2–4. **(C)** EEI cloud map at an outlet pressure of 0 Pa. **(D)** EEI cloud map at an outlet pressure of 2000 Pa. **(E)** EEI cloud map at an outlet pressure of 5,000 P.a.

Under steady inlet flow conditions, as pressure at the target outlet rises, the flow rate at that outlet decreases accordingly. Eventually, when the pressure reaches a certain point, the flow at the outlet ceases completely, resulting in zero blood flow (Figure 5A). The increase in total pressure at the target outlets (outlets 1-10) leads to a decrease in flow rates at the relevant cross-sections (CS 2-4), showing an overall linear declining trend (Figure 5B). Due to the direct proportionality between flow rate and velocity at the same cross-section, the flow velocities at each cross-section are linearly inversely correlated with outlet pressure.

Under a given pressure condition, the closer one gets to the target outlet, the greater the embolization efficiency of the injected embolic material, resulting in a larger EEI at that position. Conversely, as outlet pressure rises, the EEI gradually diminishes at the same vascular position. The cloud chart illustrating the vascular EEI is depicted in Figures 5C–E.

## 4 Discussion

The present study leverages CFD to elucidate the hemodynamic determinants of EEI during TAE procedure for hepatocellular carcinoma. Our findings demonstrate that EEI is predominantly governed by outlet pressure and vascular anatomical hierarchy rather than inlet flow rate. This challenges conventional assumptions that procedural success hinges on modulating inflow dynamics, underscoring the need for pressure-driven embolization strategies. These insights offer novel quantitative correlations to refine embolization protocols.

Most liver tumors develop an extensive network of new, abnormal, and disorganized blood vessels, a phenomenon known as hypervascularity, to support their rapid growth. These tumor vessels are often the primary targets for embolization. Tumor hemodynamics play a critical role in selecting the appropriate chemoembolization technique. However, despite the reactive dilation of tumor-feeding arteries, in computational fluid dynamics (CFD) research based on medical imaging data, accurately reconstructing, segmenting, and analyzing small tumor blood vessels remains challenging due to various factors. Therefore, we designate one or several blood vessels supplying the tumor as target outlets to simulate the macroscopic hemodynamic changes in tumor-feeding arteries during TAE procedures.

Contrary to conventional assumptions, our results revealed no correlation between inlet flow rate and EEI. This suggests that embolic distribution is governed less by flow velocity and more by vascular pressure and branching geometry. This observation corroborates prior studies (Taebi et al., 2020b; Högberg et al., 2016; Richards et al., 2012), who demonstrated that microsphere distribution in hepatic arteries depends on transient flow partitioning rather than peak flow rates. Similarly, Camobreco et al. (2025) highlighted that pulsatility-induced flow variations minimally alter particle trajectories in bifurcations dominated by pressure gradients. The decoupling of EEI from inlet flow rate implies that embolization outcomes may be more predictable across patients with varying cardiac outputs, provided pressure profiles are adequately mapped.

Prior observations revealed that flow redistribution in arterial networks is more sensitive to downstream resistance (Sun et al., 2020; Gruionu et al., 2024). In this study, the inverse linear relationship between target outlet pressure and EEI emphasizes pressure modulation as a critical therapeutic lever. As pressure increases, flow redistribution diverts embolic agents to lower-pressure non-target vessels, reducing EEI. Clinically, this underscores the importance of sequential embolization, occluding distal branches first to elevate pressure and confine subsequent embolic delivery (Horton and Adams, 2018). Our findings extend these principles by quantifying the threshold pressure required for near-complete flow cessation, offering a benchmark for procedural endpoints. In addition, incomplete embolization may arise from inadequate occlusion of collateral pathways, as residual low-pressure outlets divert embolic agents away from the target. Our results further validate the utility of pressure modulation techniques, such as balloon occlusion or vasoconstrictors, to enhance embolic delivery.

The spatial gradient in EEI, higher efficiency near the target outlet, reflects the cumulative impact of vascular branching pressure. Proximal embolization sites exhibited lower EEI due to flow dispersion across bifurcations, a finding consistent with the “vascular stealing” phenomenon. Superselective catheterization, therefore, remains paramount to maximize EEI, as demonstrated by Zhang et al. (2025) and Albrecht et al. (2021), who reported improved tumor response rates with distal embolization. Our CFD model quantifies this advantage, reinforcing the need for precision in catheter navigation.

The observed backflow patterns highlight a persistent risk of non-target embolization, particularly under pulsatile flow conditions. Embolus reflux as a major contributor to gastrointestinal complications during TAE, attributing it to transient pressure gradients during systole (van Zadelhoff et al., 2025). Our simulations extend this understanding by demonstrating that backflow arises intrinsically from vascular geometry and pulsatility. Mitigation strategies, including slower injection rates (Ren et al., 2023) or balloon-assisted embolization (Lamanna et al., 2021), may benefit from EEI-guided planning to balance efficacy and safety.

Traditional embolization endpoints, such as contrast stasis, lack the quantitative rigor needed for personalized therapy. EEI addresses this gap by providing a dynamic, spatially resolved measure of embolic distribution. This aligns with recent efforts to establish CFD-derived biomarkers for procedural planning. For instance, Roncali et al. (2020) used simulated particle trajectories to predict radioembolization dosimetry, achieving strong concordance with post-treatment imaging. Similarly, our EEI framework could guide catheter placement and embolic dosing to balance efficacy and safety.

The inverse relationship between outlet pressure and EEI suggests that pre-embolization assessment of vascular pressure, via pressure measurements or CFD, could optimize outcomes. Real-time pressure monitoring during TAE could dynamically guide embolic dosing to maintain optimal EEI. Additionally, preprocedural CFD simulations may stratify patients based on vascular pressure profiles, tailoring catheter positioning and embolic agent.

While this study provides novel insights, certain limitations warrant consideration. First, the assumption of rigid vessel walls neglects arterial compliance, which modulates flow pulsatility and pressure propagation. Second, the Newtonian blood model simplifies non-Newtonian behaviors critical in small arterioles. Third, static pressure values were used, whereas *in vivo* pressure dynamically changes during embolization due to particle lodging and vasospasm. Fourth, the model is simplistic and provides only a preliminary representation of EEI-related findings. It requires validation through more complex CFD analysis, such as simulating catheter injections and the distribution of microspheres with flow, as well as larger sample sizes. Future studies should incorporate fluid-structure interaction and non-Newtonian models to improve physiological fidelity. Future studies should integrate patient-specific viscoelastic wall properties and validate EEI correlations in multicenter cohorts. Emerging 4D flow MRI techniques could further refine boundary conditions, enhancing CFD accuracy. Lastly, *in vitro* models incorporating embolic agents are needed to validate particle-fluid interactions predicted by simulations.

## 5 Conclusion

This CFD-based proof-of-concept study suggests that target outlet pressure and vascular anatomical hierarchy may be primary determinants of embolization efficiency, while the influence of inlet flow rate appears limited. By quantifying EEI gradients and pressure thresholds, this work offers a preliminary roadmap for optimizing TAE precision-including considerations for proximal catheter placement, pressure-aware dosing, and reflux mitigation. These principles, if integrated into patient-specific computational models, could help standardize embolization protocols and potentially improve tumor response rates while reducing complications. With further development, future integration with real-time imaging and adaptive pressure algorithms may pave the way toward transforming TAE from an operator-dependent procedure into a precision oncology tool.

## Data Availability

The original contributions presented in the study are included in the article/supplementary material, further inquiries can be directed to the corresponding author.
